# Validity and reliability of a novel subtalar joint axis of rotation locator measurement device

**DOI:** 10.1186/1757-1146-7-S1-A41

**Published:** 2014-04-08

**Authors:** BH Kim, SC Lee, HD Lee, SY Lee

**Affiliations:** 1 Department of Physical Education, Yonsei University, Seoul, Korea

## Context

Inclination of the subtalar joint (STJ) in the transverse and sagittal planes may be highly associated with ankle sprain mechanisms. However, the validity and reliability of measuring inclination of the STJ axis of rotation (AoR) is not well established.

## Objective

The purposes of this study were to: 1) examine the validity of a custom made instrument (locator) to measure the STJ AoR on the basis of the STJ inclination measured by X-ray, 2) to measure the intra-tester reliability of the locator.

## Design

Cross sectional study.

## Setting

Biomechanics laboratory.

## Participants

Twenty nine healthy male (age: 22.89±9.11 yrs; weight: 77.68±18.32 kg; height: 176.16±14.16 cm) and Nine health female (age: 25±8 yrs; weight: 54.42±8.42 kg; height: 164.33±7.67 cm) subjects were recruited for this study.

## Intervention

No Intervention.

## Main outcome measures

Variables that were measured in this study were as follows: 1) Inclination of STJ AoR in the sagittal plane measured by radiographic images (Median MDXP-40 Inc, Korea) of the foot in the sagittal plane. In order to collect radiological images of the foot, subjects stood with a tandem position and the STJ was placed in neutral position. Sagittal plane inclination of the STJ AoR were further analyzed using ViewRex (TechHeim, Korea) per McClay‘s method [1]; 2) Inclination of the STJ AoR in the sagittal plane was measured by the locator; 3) Inclination of the STJ AoR in the transverse plane was measured by the locator. The anterior and posterior exit point were determined per Kirby’s method [2]. Once the locator was aligned along two points, (anterior medial and posterior lateral exit point) a Digital Mini Protractor (WWC-TE Bevel Box, USA) was used to measure inclination angle. Pearson correlation was used to analyze the relationship of validity between radiographs and the locator measuring the STJ sagittal plane inclination. Intra Correlation Coefficient (ICC) was used to analyze day-to-day reliability of the locator.

## Results

**Table 1:** Intra-test reliability about STJ sagittal plane.

	Pearson correlation(.782)		ICC(.907)	
	
	1) X-ray	2) Locator	T.1	T.2
M°±SD°	42.50±2.76	43.58±3.23	42.22±1.79	42.86±2.02

## Conclusion

The locator may be used in the clinical setting since validity verified by correlation was high and the intra-test correlation coefficient was large indicating consistent measurements. Along with the locator measurement, it is suggested that further study including motion analysis may provide more information regarding the relationship between inclination of STJ AoR and movement at the STJ.
